# Coaching in nursing: An integrative literature review

**DOI:** 10.1002/nop2.1925

**Published:** 2023-06-26

**Authors:** Corianne Richardson, Kristin Wicking, Narelle Biedermann, Tanya Langtree

**Affiliations:** ^1^ College of Healthcare Sciences, Nursing and Midwifery James Cook University Townsville Queensland Australia

**Keywords:** clinical leadership, coaching, coaching practice, developing leadership, nursing, performance development, performance improvement

## Abstract

**Aim:**

To understand the current application and benefits of coaching practice in nursing and identify potential areas for future research.

**Design:**

An integrative literature review was conducted using Whittemore and Knalf integrative review methods.

**Data sources:**

A search of the literature from Medline (PubMed) and CINHAL platform for abstracts and/or full‐text articles from 2012 to 2022.

**Methods:**

A systematic approach was used to screen and analyse the literature. Inclusion and exclusion criteria were applied, a quality appraisal of the literature was undertaken and summarised into themes. Eighteen articles were selected, two articles were different aspects of the same studies. Coaching was found to have benefits to the individual related to performance, role effectiveness, role transitions and confidence in performing in the role. The outcomes for individuals add to the overall organisational benefits with performance, support, teamwork, communication and culture.

**Results:**

This literature review sought to understand the current use of coaching in nursing and identify any gaps in the application of coaching within the discipline. Supporting, developing staff knowledge and skills and nurturing nursing staff into the profession has occurred in several ways and evolved to include coaching. Coaching provides capabilities to enhance nursing leadership, performance improvement and to provide support to staff. The findings from this literature review found a need to conceptually define coaching in nursing and the opportunity to explore the use of coaching to support both the clinical and managerial workforce (job satisfaction, intention to stay and building resilience). The benefits of coaching in nursing extend beyond the leadership level and there is opportunity to extend the operationalisation of coaching practice and coaching training within the discipline of nursing. This integrative review explains how coaching has been utilised in nursing to be a valuable tool in developing nurse leaders and clinical staff.

## 1 INTRODUCTION

Nursing is an ageing workforce, and there are many demands placed on nurses requiring them to have resilience and maintain productivity within a tighter fiscal environment (Ryan et al., 2019). Likewise, managers have increasing demands to recruit, retain and develop their workforce. These factors have led to changes in how nursing management and leadership approach the support and mentoring of their staff. These challenges do not stop at the leadership level as the performance and functioning of a nursing team is also part of the role of the shift coordinator, requiring those staff members to have skills in supporting team performance. To understand the current application of coaching in nursing, an integrative review of the literature was conducted to identify gaps and focus on future research (Whittemore & Knafl, 2005).

Coaching is gaining momentum in healthcare. Although it is less prominent than mentoring within nursing, coaching has been utilised in team building, career planning, change management and professional development (Narayanasamy & Penney, 2014). Coaching and mentoring are often referred to interchangeably in the literature, and there are varied definitions and applications of coaching within healthcare and, more specifically, the nursing domain. On review of the literature, coaching in general can be defined as an empowering partnership that is thought‐provoking and creative in process, inspiring individuals to maximise their personal and professional potential, with a positive performance outcome, that is time‐limited and focused on specific areas of development through action‐oriented goals (Bradley & Moore, 2019; Jordan et al., 2017; Westcott, 2016). The literature does distinguish some differences between the meaning of coaching and mentoring within nursing. However, the term coaching is still erroneously substituted with mentoring by some authors. This integrative review focused on the literature that was specific to coaching in a clinical nursing context.

The use of mentoring, preceptorship and clinical supervision have all held places within nursing and to support and assist the professional growth of staff. Clinical supervision is utilised widely within nursing and is a formal professional relationship between two or more people, facilitating reflective practice, exploring ethical issues and developing skills. Clinical supervision shares many similarities to coaching; however, the nature of the relationship and its separation from leadership and management is a clear distinction.

Mentoring is similar in definition to clinical supervision. However, mentoring speaks to a deeper, more long‐term relationship that focuses on support and socialisation into the profession and is not so much focused on action‐oriented performance outcomes and is distinctly different from coaching intentions (Cleary & Horsfall, 2015). Mentoring has been the traditional method to assist nurses in transitioning into the profession and developing skills, knowledge and expectations of the role (Jnah & Robinson, 2015).

## 2 BACKGROUND

Coaching has been applied within nursing as a process and tool for developing and supporting nurse leaders, developing leadership skills, reflective practice, critical thinking, performance goal setting and enhancing communication skills. Within clinical areas, coaching can be used to support staff to learn and apply new skills in the practice area. One of the tools utilised in the coaching relationship and process, is the coaching conversation. The coaching conversation can be used to support staff, and motivate and assist nurses in being focused on their own career goals. Coaching also identifies developmental needs and promotes individuals to gain meaningful challenge/feedback to help them achieve high performance and meet workplace standards of practice, which occurs through reflection, exploring actual events, goal setting and having actions to achieve those goals.

## 3 REVIEW METHODS

### 3.1 Aims

This article is an integrative review exploring coaching practice in nursing from international literature. An integrative review approach was chosen as it enables the literature to be identified, appraised and examined using a systematic method and can include both experimental and non‐experimental studies to describe the evidence concerning coaching in nursing (Whittemore & Knafl, 2005). The problem identified was understanding the current application and benefits of coaching practice in the context of coaching nurses and identifying potential areas for future research.

### 3.2 Design

The inclusion criteria for this search included all articles meeting the search terms and published between 2012 and 2022, where the topic addressed coaching in a clinical nursing setting, was primary research, a rigorous methodology was applied, and published in English. Studies were excluded if coaching was not done in a clinical setting, that is, focused on students/university/education coaching or used peer coaching. Patient health coaching was also not included in this review. Although mentoring is not the focus of this review for searching purposes, it was included in the keywords to ensure that there were no relevant articles missed that may have been using the term mentoring to refer to coaching practice.

### 3.3 Search methods

**FIGURE 1:**
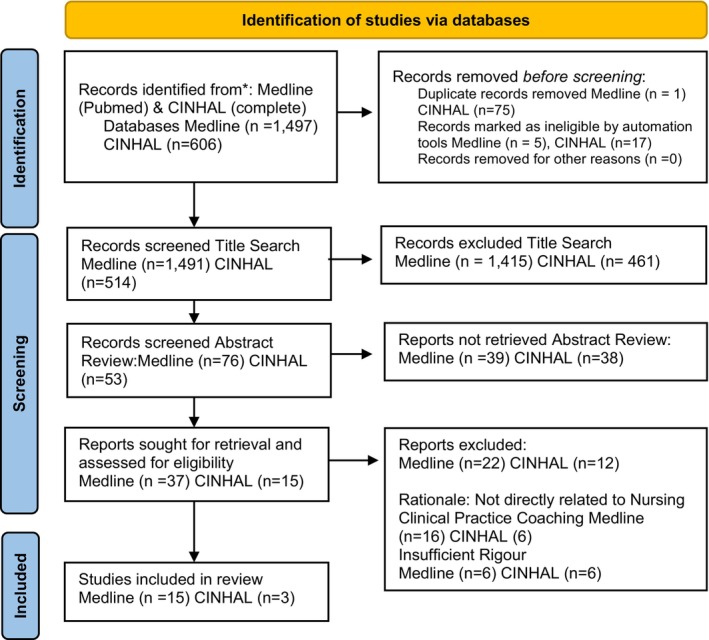
Prisma flow diagram coaching studies. *PRISMA flow diagram (adapted) of the study selection process. Page et al. (2021).

Medline (PubMed) and CINAHL Platform were searched for abstracts and/or full‐text articles. The search terms used in CINAHL were ‘nursing AND coaching AND mentoring’ SmartText search, PubMed ([nursing] AND coaching) AND mentoring ‘subheading’ or ‘all fields’ (MeSH terms). Duplicates were removed by endnote, and manually removed across databases. A title and abstract screening was conducted, resulting in 42 articles that underwent a detailed review against the inclusion and exclusion criteria. Reference lists were hand‐searched to include significant articles that may not have been located through the above systematic searching. Please see Figure 1 Prisma Flow Diagram Coaching Studies. The PRISMA checklist was used as a guide to full reporting of this integrative review (Moher et al., 2009).

### 3.4 Search outcome and quality appraisal

Forty‐two articles were included in the appraisal for methodological rigour, topic relevance and meeting the inclusion/exclusion criteria for the topic of coaching in nursing, resulting in a final data set of *N* = 18 articles. Any discrepancy about the selection of studies were resolved by consulting with authors (NB and KW). CASP systematic review checklists were used to critically evaluate the literature as it is a set of multiple checklists for different study designs (Critical Appraisal Skills Program, 2019). All authors assessed the quality and relevance of the studies and discussed the appraisal decisions. Those articles not included where articles were the focus was not on coaching in clinical nursing and articles that did not employ a research method. Agreement was reached for inclusion of the final data set by all authors.

## 4 RESULTS

These results described coaching in clinical nursing contexts and reported on coaching for improving staff performance and providing support in professional development. There were 16 independent studies, two studies published different aspects of their findings (*N* = 18 papers). Five of these studies were intervention studies and one of these was a pilot intervention study (*n* = 6), Four were descriptive exploratory studies (*n* = 5) with two publications of different elements of the study findings, and three were evaluation studies, (*n* = 3) with seven of these 12 studies using a mixed methods approach with surveys and semi‐structured interviews. One was a grounded theory study (*n* = 1), one quality improvement study (*n* = 1) and one pilot randomised control trial (RCT) study was included (*n* = 2), which had two articles published: one reported the feasibility, and the other reported on the effectiveness of the intervention. Several articles (*n* = 13) described coaching interventions as part of leadership development programmes for middle managers or focused on nurse leaders and advanced practice nurses. Other coaching applications were in acute environments to support educational interventions.

The impact of coaching in these studies had effects at both the individual and organisational levels, with common themes emerging across the studies. The reviewed studies' themes included: performance, communication and support at an individual level. There were also some impacts from the performance outcomes and discussions with staff that, in turn, positively affected the organisation. The literature review process also highlighted the need for better precision in the conceptual definition of coaching as it applies within a nursing context. Table 1: Synthesis of the Literature Reviewed and Key Findings.

**TABLE 1:** Synthesis of literature reviewed and key findings, in chronological order.

Author (year)	Study design	Data collection	Sample description	Key findings
Cummings et al. (2014)	Pilot – Intervention study	Quantitative—pre/post surveys Qualitative—focus groups	*N* = 21 participants with *n* = 12 respondents to both pre‐ and post‐survey	Intervention teaching managers coaching and communication skills. Barriers to coaching: Time, staff unwillingness to be coached, little interest in learning new things or updating skills. The coaching relationship requires staff agreement, trust and respect
McNamara et al. (2014)	Evaluation study	Qualitative study—using a grounded theory approach. Multiple methods	Programme participants (*n* = 50) and coaches/mentors (*n* = 20)	Coaching assisted communication and responding to everyday problems. Participants felt motivated and pushed by the coaches. Coaches saw the benefit in coaching in leadership training. Coaching is based on the client's ‘agenda’. The threat to effective coaching was identified when personal and emotional issues emerged
Rafferty and Fairbrother (2015)	Grounded theory study	Qualitative—in‐depth interview Reflective journal	*N* = 20 senior nurses and midwives	Pre‐existing perceptions can impact on uptake and use of coaching skills and having the courage to change and/or try the skills in practice. The credibility, support and trust of the coach are essential to the relationship. Organisational support is essential to the ongoing use of these skills. For some, it is a complete change in management style. There needs to be an opportunity to practice and receive feedback
Westcott (2016)	Descriptive exploratory study	Mixed methods	*N* = 21 Nurse managers, coaches and directors of nursing	This article is only reporting on the qualitative interviews. NMs used coaching to becomes self‐aware, self‐reflective and improve potential. Following coaching, nurse managers gained increased resilience, confidence and coping strategies. The increased confidence resulted in perceived improvement in team management and cohesion
Anderson et al. (2017)	Intervention study	Mixed methods	*N* = 428 bedside nurses and *n* = 8 nurse leaders	Nurses reported statistically significant^a^ improvement in their level of skill post‐workshop compared to survey completed before the workshop. Enhanced the performance of nursing staff and embedded learning of new skills related to palliative care communication
Jordan et al. (2017)	Pilot RCT	Mixed methods	*N* = 17 dyads CNAs/residents	The control group received 25‐min traditional video training and the experimental group received the traditional video training, plus three coaching sessions over 4 weeks. This pilot RCT supported coach training as a method to improve appropriate use of LoA strategies
Le Comte and McClelland (2017)	Evaluation study	Mixed methods	N = 71 participants (24.4% response rate) and N = 15 Senior staff supporting the programme	Participants described: improved communication skills, emotional intelligence made positive changes to a more supportive team environment; organisational culture better aligned to the organisations' values. Several key benefits identified: work/life balance, listening skills, understanding the bigger picture and performance management. Improved relationships with other senior staff /direct line manager. Challenges in implementation included lack of opportunity to coach and decreased capacity due to time scheduling
Cummings et al. (2018)	Intervention study	Quantitative survey data	*N* = 333 completed surveys	Coaching conversations negatively impacted job satisfaction. Coaching conversations were influenced by feedback processes. Resonant leadership significantly predicted manager support, which in turn predicted coaching conversations
Cable and Graham (2018)	Intervention study	Mixed methods	*N* = 116 nurse managers surveys and *n* = 11 In‐depth interviews	Positive impact, beneficial relationship, enjoyment, support, stretch, insight and usefulness. In all criteria, perceptions by coachees were substantially positive
Jordan et al. (2018)	Pilot RCT	Mixed methods	*N* = 17 Certified nursing assistants (CNAs)	Coaching helped embed skills taught to assistants during skills training. Coaching training intervention is feasible. There was improvement in appropriateness of Level of Assistance (LoA) use and independence dressing scores between pre‐ and post‐test but not in the traditional training. The coaching intervention training was reported as feasible with retention rates of 89.47% CNAs and 85% residents
Yusuf et al. (2018)	Quality improvement project	Mixed methods	Not clearly stated	A significant improvement in relationships was reported, and the results sustained at 1 year. There was a statistical significance in perceptions of teamwork and overall improvement in departmental culture and communication. Changes to perceptions of competence as a team did not change until the second assessment, indicating that it takes time for change at this level to be seen
Bradley and Moore (2019)	Descriptive exploratory study	Qualitative: semi‐structured interviews	*N* = 11 coaches providing coaching to nurse managers (NMs)	Coaching assists in supporting NM's and helping in their role effectiveness. Coaches described the NM's sought out coaching to help with conflicts with both staff and higher level leaders. Coaching contribution, two categories emerged: fostering self‐efficacy/self‐awareness and providing multilevel positive impact
Waldrop and Derouin (2019)	Evaluation Study	Cross‐sectional survey design	N = 83 Advanced practice nurses	Dissatisfaction with the coaching was related to scheduling and lack of commitment by other coaches. A good match of coach and coachee was important by participants. Coachees agreed their coach provided encouragement, feedback and guidance. Coaches who are excellent listeners, proactive in troubleshooting and demonstrate respect for the group are ideal coaches
Bradley and Moore (2020)	Descriptive exploratory study	Qualitative: semi‐structured interviews	*N* = 11 coaches providing coaching to nurse managers (NMs)	Expands on the previous article published in 2019. This article focuses on outcomes for the coaching relationship and selection of a coach. Competence, Coach–coachee fit and trial session were three emerging categories. Two themes emerged for ground rules of coaching relationship: Outlining the nuts and bolts of the relationship and confidentiality
Ali et al. (2021)	Descriptive exploratory study	Quantitative survey	*N* = 311 nurse responded. Sample from 38 hospitals (public and private)	Managerial coaching has a significant association with organisational identification, psychological ownership and workplace well‐being. Managers who engage in coaching behaviours such as providing consistent feedback, enhancing their learning abilities, openly communicating and facilitating goal achievement see benefits in employees
Douglas and MacPherson (2021)	Intervention study	Single subject, multiple baseline design. Pre‐ and post‐testing	*N* = 9 certified nursing assistants (CNAs) and *N* = 7 dementia residents	Six‐session weekly strategy (15 min duration) to each CAN designed to support self‐perceived knowledge (SPK), self‐efficacy and practice positive communication behaviours. Participants demonstrated an increase in SPKE measure, however, pre and post one‐tailed paired samples were not statistically significant. Improvements were seen in communication and responsive behaviours of people with dementia
Moura et al. (2020)	Descriptive‐analytical study	Survey	*N* = 221 – *n* = 210 nursing technicians and *n* = 11 nursing coordinators	The practice of coaching leadership in self‐perception of coordinators and perception of nursing technicians. Dimension of ‘giving and receiving’ feedback had the highest mean and high score among the coordinators interviewed. The second highest was ‘communication’ domain. Nurse technician's responses saw an inversion of the order. A statistically significant indirect correlation was observed between giving and receiving feedback and training time, the greater the practice of giving and receiving performance information. It appears that feedback allows the leader to expand and improve his self‐perception, in addition to contributing to the learning of those involved
Spiva et al. (2021)	Quasi‐experimental pre/post survey design	Programme Evaluation—using survey	*N* = 46 nurses	Training Intervention: focused on resiliency, essential nurse manager orientation and strength‐based coaching. There was significant difference in pre‐ and post‐survey in resiliency, transformational leadership, leadership effectiveness and leadership satisfaction scores. Findings support that training in transformational leadership behaviours and resilience improves both qualities in frontline nurse leaders

^a^ Statistically significant refers to a *p* ≤ 0.5.

### 4.1 Purpose of coaching

The effect of coaching on the individual fell into the following key areas: performance and role efficacy, communication and support. Many current and emerging clinical leaders feel inadequately prepared for their roles (Bradley & Moore, 2019, 2020; Westcott, 2016). Coaching can assist with role transitions and identify critical skills for development (Spiva et al., 2021; Westcott, 2016). The studies in this literature review focused on leadership development were all pitched at the Nurse Unit Manager (NUM) level. Participants in the 18 studies were nurse managers/advanced practice nurses (*n* = 8), bedside nurses (*n* = 2), a mix of nurse managers and bedside nurses (*n* = 4), nursing aides (*n* = 1) and external coaches (*n* = 1). The country of origin for these studies was predominately the United States (*n* = 12) and the United Kingdom (*n* = 3).

As shown in Table 2: Use of coaching, there were applications in how coaching was integrated into the studies reviewed. There was no consistent definition of coaching provided in these studies. Coaching was utilised to support educational interventions and embed these into practice. Coaching was also used to train nurse managers as coaches to assist in their management role and provide coaching to support and develop those nurse managers. Finally, coaching was used to explore mentoring in practice and to change workplace culture. This synthesis notes the impact of coaching on self‐development, which included self‐efficacy and self‐awareness. The increased self‐awareness led to a change in focus on self‐development but also interactions with others (Cable & Graham, 2018; Le Comte & McClelland, 2017). The impact (outcomes) of coaching was also dependent upon the application of coaching process Table 3: Synthesis of Themes Process and Outcomes of Coaching in Nursing.

**TABLE 2:** Use of coaching.

How coaching was used in integrative review studies (*N* = 16)	
To train nurse managers to become coaches/coach leaders (Cable & Graham, 2018; Cummings et al., 2014, 2018; Le Comte & McClelland, 2017; Moura et al., 2020; Rafferty & Fairbrother, 2015; Spiva et al., 2021)	7
To support educational interventions for clinical staff (Anderson et al., 2017; Douglas & MacPherson, 2021; Jordan et al., 2017, 2018)	3^a^
To develop nurse managers (Bradley & Moore, 2019; Bradley & Moore, 2020; McNamara et al., 2014; Westcott, 2016)	3^a^
To develop Advanced practice nurses (APN) (Waldrop & Derouin, 2019)	1
To foster positive workplace culture in nursing (Ali et al., 2021; Yusuf et al., 2018)	2

^a^ Two studies with two publications on different aspects of the studies.

**TABLE 3:** Synthesis of themes: process and outcomes of coaching in nursing.

	Process of coaching		Outcomes of coaching							
	Leadership development	Coaching skills	Clinical skills	Development of self	Performance/Role effectiveness	Communication skills	Culture^a^	Job satisfaction	Trust	Support^a^
				^b^ Self‐efficacy Self‐awareness leadership skill	^b^ Improved performance Improved leadership capability	^b^ ‘Coaching Conversation’ ‘Communication Effectiveness’				
Cable and Graham (2018)	✓			✓	✓	✓	✓	✓	✓	✓
Le Comte and McClelland (2017)	✓			✓	✓	✓	✓		✓	✓
Waldrop and Derouin (2019)	✓			✓	✓	✓			✓	✓
McNamara et al. (2014)	✓				✓	✓		✓		✓
Westcott (2016)	✓	✓			✓	✓	✓		✓	
Spiva et al. (2021)	✓	✓		✓	✓					✓
Cummings et al. (2014)	✓	✓			✓			✓		
Moura et al. (2020)	✓	✓		✓		✓				
Rafferty & Fairbrother, 2015)		✓		✓	✓	✓	✓		✓	✓
Ali et al. (2021)		✓			✓	✓	✓			✓
Bradley and Moore (2019)		✓		✓	✓	✓	✓			✓
Cummings et al. (2018)		✓		✓	✓		✓			✓
Yusuf et al. (2018)		✓		✓	✓	✓	✓			✓
Bradley and Moore (2020)		✓		✓	✓	✓			✓	✓
Anderson et al. (2017)		✓	✓		✓	✓				✓
Jordan et al. (2018)		✓	✓		✓					
Jordan et al. (2017)		✓	✓		✓					
Douglas and MacPherson (2021)			✓	✓	✓	✓				

^a^ In both sub‐themes of support and culture additional outcomes emerged: well‐being and positive relationship building.

^b^ Sub‐themes identified.

### 4.2 Coaching improved role effectiveness and performance

Coaching for performance occurs through a focused process that develops between the coach and coachee. The literature reviewed described varied aspects of the coaching relationship using internal and external coaches. The 18 articles reviewed had performance as an outcome or cited by participants. Improvement in skills by the transfer of knowledge directly impacted performance in practice by development in role effectiveness through increased self‐confidence and achieving personal goals (Bradley & Moore, 2019, 2020; Rafferty & Fairbrother, 2015). Bradley and Moore (2019) engaged the use of coaches to work with nurse unit managers (NUM's). They highlighted the challenging role of NUMs to perform their positions due to the span of their control (the enormity of the job). A total of 100% of coaches in this study sought assistance with role development and reported they gained a better sense of themselves and their capability. As part of work performance, there was a close association to role effectiveness (Bradley & Moore, 2019).

Cable and Graham (2018) reported coaching had a positive impact on participants, pushing participants to perform. Nurse managers found value in the 1:1 coaching session through the opportunity to reflect and identify what to change in themselves and how they impact others. This reflection assisted in building confidence in their leadership style (Cable & Graham, 2018). Similarly, Waldrop and Derouin (2019) found that advanced practice nurses developed their leadership skills and project deliverables through coaching dyads in an evaluation study. Nurses valued the feedback process as necessary for their performance improvement. Ali et al. (2021) in their descriptive/exploratory study of 311 nurses also found that managers who engaged in behaviours such as providing feedback, had enhanced learning experiences, improved communication and facilitated goal achievement.

In an evaluation study of a leadership development programme, Le Comte and McClelland (2017) reported that participants described an increased ability in a range of areas, including performance management, confidence and ability to lead. Likewise, McNamara et al. (2014) reported that coaching assisted in motivating and pushing the participants, with one participant describing how the intervention had helped her build her communication skills.

Jordan et al. (2017) conducted a randomised control trial of coaching to support an educational training intervention. This study showed a notable improvement in staff performance of those staff who received coaching compared to the control group. This study found that with coaching the application of the skills taught were implemented more appropriately in the clinical setting (Jordan et al., 2017, 2018). Anderson et al. (2017) also utilised a training skills intervention for nurses in communication to improve the staff response to palliative care needs, and their findings also noted a performance improvement. There were noted benefits for study participants where teaching or skills training is combined with coaching support.

Several studies found that coaching supports performance through improved confidence and critical thinking. There were also noted benefits at both individual and team level. Similarly, in their intervention study of coaching to support certified nursing assistant (CNA), Douglas and MacPherson (2021) found that a 6‐week coaching strategy resulted in positive changes to self‐perceived knowledge and impacted on positive communications behaviour. This intervention also impacted on decreasing the negative responsive behaviours of people with dementia, due to the changes in the CNA's approach. Coaching was linked to active learning that leads to improved performance, and nurses have found they develop their skills, knowledge and confidence with the support of coaching (Douglas & MacPherson, 2021; McNamara et al., 2014; Waldrop & Derouin, 2019).

In Cummings et al. (2018) study on coaching conversations, the researchers' aim was for their coaching intervention to develop leadership style and they used coaching conversations to influence staff knowledge, use of learning and performance (Cummings et al., 2018). Staff also needed some assistance with role development as they transitioned into the nurse unit manager or leadership positions. In a descriptive analytical study of 210 nursing technicians and 11 nursing coordinators, Moura et al. (2020) found that feedback allowed leaders to expand and improve self‐perception, which in turn contributed to learning of those involved. Two other studies reviewed also acknowledged improved performance specifically related to the area of communication at both the individual and team level (Rafferty & Fairbrother, 2015; Westcott, 2016). Yusuf et al. (2018) conducted a quality improvement study of nurses working in a neurosurgical ICU and found that the coaching programme was an effective method for improving nurse–physician working relationships, with a significant change in nurses' perceptions of teamwork, with open communication being statistically significantly improved at 5 months and 13 months, showing that this change could be sustained over time. The varying lengths of the coaching sessions emerged as an area requiring further research and evaluation.

### 4.3 Coaching improved communication

Communication within a nursing context is essential to the provision of high quality and optimum patient outcomes (Bramhall, 2014). Nurses use communication to relay and interpret information between each other, medical staff, caregivers, family members and patients. The literature review found that there was an impacted on communication at the individual level through individual skill development and the ability to manage conflict (Le Comte & McClelland, 2017; Rafferty & Fairbrother, 2015). Le Comte and McClelland (2017) found that managers transformed the way they framed discussions and changed the language they used from reactionary and negative to open and supportive. In the 18 articles reviewed, 12 found some beneficial relationship or outcome related to communication. Anderson et al. (2017) purported that communication training, combined with coaching, helped bedside nurses feel more skilled in utilising palliative communication strategies. However, the study only sought participants' self‐reflections and not the family's or carer's experiences, which was a limitation to the study.

In their grounded theory study of 20 senior nurses who received coaching and leadership training, Rafferty and Fairbrother (2015) found staff implemented coaching skills into a variety of situations including conflict resolution, clinical discussions, office drop‐ins and complaints investigation. Communication regarding feedback around performance can assist with nurses identifying potential areas to focus their development. Cummings et al. (2018) was the only study that reported a negative impact of coaching conversations and noted that one‐on‐one coaching conversations may be difficult for staff who are not used to participating in this type of communication. Furthermore, this study was the only study of the 18 reviewed that identified a negative experience with coaching, and participants also described a negative impact on job satisfaction. This finding potentially was related to how feedback was given and the staff exposure to this level of input within the clinical setting. Suppose staff are not familiar with receiving feedback or it is delivered negatively, in that case, this experience can be received with mixed feelings and perceptions, thus negatively impacting how coaching is perceived and experienced.

In a study by Bradley and Moore (2019), coach participants described that nurse managers sought out assistance from the coach to deal with conflict situations. Westcott (2016) claimed that coaching led to a reduction in conflict because problems were identified quickly and managed more effectively through effective communication. These studies concluded that communication effectiveness and the ability to control the process of conflict resolution and performance feedback is a skill that is not common to all people and is one that developing leaders and existing leaders seek to master (McNamara et al., 2014; Westcott, 2016).

### 4.4 Coaching improved resilience

Having the opportunity to explore work/life balance and to be less critical of self, develop presence and leadership courage were reported as benefits from coaching (Bradley & Moore, 2019). Coaching was seen to have restorative, normative and formative effects by supporting clinicians during times of stress or conflict, providing honest feedback and opportunity for reflection on current practice and assisting with identifying learning opportunities, skill enhancement and professional development (Boyer et al., 2018;Bradley & Moore, 2019; Cummings et al., 2018). Westcott (2016) also reported those nurses participating in their study described increased resilience, increased confidence and better coping mechanisms. In their recent study of 46 nurses, Spiva et al. (2021) evaluated a training intervention in leadership and strengths‐based coaching as an effective evidence based modality to improve leadership style and resiliency. The findings supported that training in transformational leadership behaviours and resilience improves both qualities in frontline leaders.

### 4.5 Impact on workplace culture

The impact on workplace and culture were mentioned in a few studies. Two studies found an impact of coaching on job satisfaction (Bradley & Moore, 2019; Cummings et al., 2018). Ali et al.'s (2021) descriptive exploratory study of 311 nurses focused on managerial coaching in nursing and their findings supported the hypothesis that coaching behaviours, such as feedback and open communication, impacted on the employee, with significant association to organisational identification, psychological ownership and workplace well‐being. (Bradley & Moore, 2019) described multi‐level impacts with happier staff, retention of staff and better patient satisfaction. Cummings et al. (2018) mentioned work context as impacts on staff job satisfaction and coaching conversations did not result in a positive outcome for staff in this study. The most positive effects were described in studies of nursing leaders who reported transformations within them to deliver enhancement in individual, team and service performance (Cable & Graham, 2018; Le Comte & McClelland, 2017; McNamara et al., 2014). Yusuf et al. (2018) and Moura et al. (2020) found that coaching outcomes improved relationships led to enhanced workplace performance, and self‐empowerment both positive aspects of workplace culture. Rafferty and Fairbrother (2015) reported that optimism about work challenges and emerging unit culture change occurred. The researchers also raised the important issue around the need for organisational support for a culture of coaching, and such support includes the necessary time release for participants to engage in coaching.

### 4.6 Challenges encountered in delivering the intervention of coaching

Several challenges were identified from a review of the literature. One of these was the sustainability of coaching with participants citing difficulties with the opportunity to mentor and coach others, and a lack of capacity: having time and confidence in implementing the strategies taught (Le Comte & McClelland, 2017). Rafferty and Fairbrother (2015) found that some of the coaching interventions were not always used appropriately, and this inappropriateness was linked to negative participant experience, role dissonance and decreased self‐efficacy and increased belief in others to find the solutions. There was discussion around the issues of having internal coaches or external coaches, with some studies providing mixed reports about the distinction of coach selection from participants. Westcott (2016) recommended the coach should be external to the line manager as some staff have issues with their line managers. Similarly, in other studies, participants were more comfortable with external coaches in discussing their concerns, developing trust and not being swayed by internal politics (McNamara et al., 2014). The use of coaches external to the organisation has been reported as helpful in maintaining objectivity and generating innovative ideas (Waldrop & Derouin, 2019).

The choice of coach ultimately should be given to the staff member selecting the coach, as trust is an important component in the coaching relationship. Success was found where there was support from senior managers to implement the programme and where staff gained additional support in developing their skills through their ongoing coaching once they had returned to the workplace (Rafferty & Fairbrother, 2015). Waldrop and Derouin (2019) also found that there were reported dissatisfaction with the coaching programme when there were cancellation and rescheduling, with some issues also noted around commitment by coaches to attend.

### 4.7 Strategies that assisted with the implementation of coaching

Rafferty and Fairbrother (2015) found that support provided to staff to develop their coaching skills had a catalytic effect on successful uptake of the skills. Providing individual and group opportunities to practice coaching skills was affirmed to be supportive of participants. Further, Bradley and Moore (2020) highlighted best practices for working with a coach, emphasising the key categories of: the competence of the coach, coach–coachee fit and offering a trial session. Matching coaches and coachees within the coaching programmes were also seen to have positive benefits to the success of the relationship (Boyer et al., 2018; Bradley & Moore, 2020; Waldrop & Derouin, 2019). Engagement and participation also received a more favourable response when staff attended introduction sessions as part of the lead up to leadership and coaching training (Boyer et al., 2018). Establishing trust and reassuring confidentiality were considered key issues in findings from Bradley and Moore (2020). The importance of ensuring that personal problems were addressed in advance of commencing coaching was emphasised by having clear boundaries around the coaching relationship (McNamara et al., 2014). Bradley and Moore (2020) also identified key attributes of nurse managers that assist in the success of the coaching relationship, such as being open to transformation and being attentive to self. The ability for nurses to engage in the coaching relationship does require openness and willingness to change/develop (Bradley & Moore, 2019, 2020). Managers also need to be able to build and maintain healthy relationships with their subordinates (Ali et al., 2021).

## 5 DISCUSSION

The findings from this integrative review helped to identify critical areas where coaching is utilised, and the benefits that are seen consistently across these studies included improvements in performance, communication and support. Each of these areas act to interrelate in the way coaching is applied, in the way coaching is experienced and in the outcome of the coaching process. There are key aspects to successful implementation of coaching within the workplace, these include the coaching relationship, the use of coaching conversations and feedback, and the focus on learning and development. Coaching has both organisational and individual benefits that support leadership development, relational dynamics and personal growth.

### 5.1 Coaching and performance

Coaching was seen to have an impact on the performance of staff. This was seen in how coaching affected the development of skill, knowledge and transferring this learning into practice. Communication was positively impacted by supporting individuals to reflect on their skills and influence and supporting new ways to work with others. Support was demonstrated through gaining assistance with problem‐solving, listening, receiving feedback and managing conflicts. Coaching was also seen as a tool to assist in learning and development and applying new skills (Cummings et al., 2014; Douglas & MacPherson, 2021; Jordan et al., 2017).

### 5.2 Coaching relationship and trust

Several studies discussed the importance of the coaching relationship and trust within that relationship. There was a varied application of how coaching was implemented across studies from having coaches who were external to unit or organisation, internally trained coaches who are nurses or external coaches who may or may not be nurses. Some studies also included a train the trainer approach where nurses (leaders) received coaching and undertook training to become a coach or use coaching as part of their leadership approach. McNamara et al. (2014) describe mixed views by participants around the assignment of mentor/coach. There were some common factors mentioned across studies. These included the need for confidentiality and being honest in the process and having credibility (McNamara et al., 2014; Rafferty & Fairbrother, 2015). The need for psychological safety was also highlighted, this is where some studies made a preference to having an external coach (Rafferty & Fairbrother, 2015). This psychological safety was specifically mentioned by Waldrop and Derouin (2019), where nurse managers in this cross‐sectional survey study evaluating coaching circle experience across four cohorts from 2013 to 2017. Although coaches were assigned, the participants reported that important factors included values, standards and the need to have a coach who intrinsically had the same ethics and values, but who also had an external outlook. Westcott (2016) similarly described the experience of participants related to encouragements, positive feedback and constructive criticism being supportive. Most of the studies were not long‐term studies, so there is a need to have robust evaluation of the impact of coaching for staff across a longitudinal timeframe.

Coaching is used in a range of environments, and there have been similar findings in both the business and psychology literature that support the use of coaching for performance and teamwork. The reported outcomes and experience of coaching for nurses are not dissimilar to other professions. Psychology has helped to inform coaching practice in nursing using positive psychological principles, drawing on elements of humanity such as happiness, wisdom, creativity and strength (Biswas–Diener, 2020). Benefits within the business/psychology literature included generating improvements in individual performance, increased openness to personal learning and development and helping to identify solutions to specific work‐related issues (Atad & Grant, 2020; Grover & Furnham, 2016; Wilson, 2004). These benefits were also seen in nursing practice in several of the studies reviewed here.

### 5.3 Self‐awareness and self‐efficacy

Coaching is helpful in the development of staff and in improving performance, specifically around assisting staff in gaining a better understanding of themselves and their capabilities. This aspect of self‐efficacy is essential in performance as the confidence in oneself to do the job assists in shaping the belief and behaviour required to produce specific performance attainments (Barrera et al., 2020).

### 5.4 Development of self

Coaching also supports the learning process, focusing on both the physical and social aspects of learning, and tackling the barriers (mostly psychological) that can affect the learning process or experience. Coaching as a supervisor or leader has been likened to that of a sports coach; it enables focus and dialogue to help motivate and prepare individuals to work hard and play as a team (Shaneberger, 2008). The communication benefits identified from coaching were key to building healthy work environments, and a way to engage employees (Korth, 2016). Korth (2016) also recognised that there is growing evidence that a healthy work environment impacts positively on staff satisfaction, retention, improved patient outcomes and organisational performance.

### 5.5 Resilience and coaching

Participants in Bradley and Moore (2019) study reported benefits that would assist in building a more resilient practitioner, which was also found in Westcott (2016) study where their participants described increased resilience. Cable and Graham (2018) found benefits examples given by a coachee to exemplify the positivity and resilience aspects included ‘valuing the protected 1:1 coaching time’ and ‘having the opportunity to see what to change about myself’ and learn of the impact the individual has on others. Spiva et al. (2021) supported the effectiveness of a coaching programme for nurse managers to improve transformational leadership skills and resilience. The findings in these studies support the psychology literature where case study reports of increased ability to handle stress and conflict and greater productivity were noted (Bartlem et al., 2018; Grant, 2001). However, it would be valuable to have further investigation into the impact of coaching behaviours by leaders and frontline clinical nurses in the development of resilience.

### 5.6 Communication skills and conversation

Communication has been identified as a fundamental element of a healthy work environment, and coaching can play a role in developing communication effectiveness, conflict resolution and specific coaching conversations related to workplace performance and self‐reflection (Bradley & Moore, 2019; Korth, 2016; Welp et al., 2018; Westcott, 2016). While Cummings et al. (2018) reported some negative outcomes with the use of coaching conversations, the researcher reports that they found emotionally intelligent leadership practices by formal nursing leaders had a direct and positive relationship with staff job satisfaction. Giving and receiving feedback and communication was also seen as a positive outcome in a coaching leadership model (Moura et al., 2020).

### 5.7 Limitations

The literature reviewed used search terms that included mentoring and coaching, as there is ambiguity within the literature defining coaching in nursing. There was conflation of the concepts of coaching and mentoring seen in some of the grey material excluded from this review. Coaching is still an emerging area of research within nursing, and the implementation of coaching as both an educational/training support measure and a leadership development strategy has not assisted in creating a clear delineation of coaching in a nursing context. The results of the study by Rafferty and Fairbrother (2015) provide insight into the learning process and use of coaching leadership training for nurse managers that is worth consideration for implementing coaching in practice.

There were varied methodologies used in the studies reviewed. Most of the participants in these studies were nurse managers, with a small sample size that would impact the generalisability of the findings. The randomised control trial by Jordan et al. (2017) had some issues with recruitment due to the structure of the intervention and consent issues with consumer participants delaying the start of the study. There was also data contamination with the control group talking with the experimental group about the intervention. Le Comte and McClelland (2017) study had low response rates, and there was no description as to how the researcher treated the qualitative data. There was also no clear understanding of how the researchers managed potential bias, as some senior staff participating in this study would have had a vested interest in the programme's success. The studies reviewed for this article do not discuss any long‐term effects, as the majority of the research was conducted within a 12‐month to 2‐year time frame. Therefore, long‐term sustainability is not able to be discussed.

The studies in this review where nurses were trained as coaches did not describe the content of their coaching training, so it is difficult to determine if there were vital universal elements included in these training programmes, or their key focus. This lack of provided curriculum detail highlights an issue around the unknown and varying quality of training, and the need for policymakers to define the competencies and standards of coaching and coach training for nurses in both leadership and clinical roles.

### 5.8 Practice implications

Several recommendations impacting on practice emerge from this literature review. These cover three specific areas: coaching practice, coach training and future coaching research. Performance in a workplace and as a nurse is impacted by a range of factors both internally and externally. However, to sustain high performance, there needs to be an investment in the ‘self’, and this investment is linked to motivation, focus and self‐awareness. Setting performance goals and learning and development plans are also integrated components of working in a hospital setting. Receiving feedback from managers and supervisors, and the relationship and conversations in which that feedback occurs, are all central components to the experience and performance of staff.

#### 5.8.1 The practice of coaching

Not all staff are comfortable in the coaching process; this was evident in the findings by Cummings et al. (2018). This highlights a key aspect of consideration when implementing coaching within the workplace and the need to consider how coaching process is taught and the use of conversation as a managerial tool. Coaching practice requires educational preparation and a clear understanding of coaching theory, process and skills. The support of coaches in how they utilise coaching in their practice as leaders and clinicians are essential to successfully implementing coaching programmes within nursing. It is recommended that coaching programmes and coaches are provided with specific support around the use of their coaching skills, understanding the formal phases of coaching for formal coaching sessions and coaching in the moment.

#### 5.8.2 Coach training

There is a place for coaching in nursing practice, and the positive benefits of coaching for both managers and clinical staff are evident. The content of training for staff to use coaching is an area that is not well documented, and the literature reviewed did not provide detail on the content of those programmes. There are also varied approaches used in coaching that draw on psychology's domain, such as cognitive behavioural and solution‐focused coaching. Understanding how these theoretical underpinnings influence the coaches and coachees within a nursing context would be valuable. It would be recommended that research be undertaken to demonstrate and evaluate the effectiveness of coach training for nurses. Staff who are not familiar with coaching may find it to be confronting and challenging. Therefore, developing trust within a coaching relationship is an essential distinguishing attribute of coaching practice. It is recommended for any coaching education that consideration is given to staff preparation to understand what coaching is and how coaching conversations are utilised, and the critical elements of the formation/development of the coaching relationship.

#### 5.8.3 Future coaching research

It is worthwhile to explore the relationship between coaching and resilience for nurses practising on the frontline to provide support, improve performance and increase workforce retention. There would be an opportunity for research with a longitudinal approach to explore further the impact of coaching over time and any changes or outcomes that may benefit from a more extended evaluation period. There is an opportunity for further research in understanding the factors that foster the uptake of coaching, which will support successful implementation within a nursing context.

## 6 CONCLUSIONS

Organisations need to provide Nurse Unit Managers and Clinical Nurses with the proper leadership development and skills to improve individual and team performance at the clinical frontline. Communication has been a fundamental element of healthy work environments (Hartung & Miller, 2013). Relationship building and maintaining positive workplace relations assist in keeping employees engaged and motivated (Barrera et al., 2020). Coaching provides tools that can be utilised for the support and performance of staff members.

Further research into how coaching programmes can assist clinical leaders in performing their roles is essential. The need to equip both the nurse leader and shift coordinators with skills and tools that help in creating healthy workplaces is critical. There is a clear gap in the research focusing on the clinical frontline leadership roles within nursing, such as the Clinical Nurse. Clinical Nurses/Shift Coordinators are required to think on their feet using situational knowledge and expertise and would thus likely benefit from coaching as well. The use of coaching may assist in improving professional identity, job satisfaction and intention to stay in the profession. There is a requirement for health services to focus on leadership programmes that create sustainable change, build nurses' resilience and provide skills that help to promote high‐level work practice. There is a place for coaching within nursing, and there is an opportunity to explore further the benefits, challenges and ways in which coaching can be implemented to support nurses in their development and practice.

## CONFLICT OF INTEREST STATEMENT

None.

## ETHICS STATEMENT

None.

## Data Availability

Data sharing not applicable to this article as no data sets were generated or analysed during this study.
